# Plain Water Intake and Association With the Risk of Overweight in the Chinese Adult Population: China Health and Nutrition Survey 2006–2011

**DOI:** 10.2188/jea.JE20180223

**Published:** 2020-03-05

**Authors:** Xing-Bing Pan, Hui-Jun Wang, Bing Zhang, Ying-Li Liu, Su-Fen Qi, Qing-Bao Tian

**Affiliations:** 1 Department of Epidemiology and Statistics, Hebei Key Laboratory of Environment and Human Health, School of Public Health, Hebei Medical University, Hebei, China; 2 National Institutes for Nutrition and Food Safety, Chinese Center for Disease Control and Prevention, Beijing, China

**Keywords:** plain water, overweight, obesity, dose–response, CHNS

## Abstract

**Background:**

The prevalence of overweight is increasing dramatically worldwide. The aim of our study was to investigate the association of plain water intake (PWI) with the risk of new-onset overweight risk among Chinese adults.

**Methods:**

A total of 3,200 adults aged 18–65 who were free of overweight at baseline were enrolled from China Health and Nutrition Survey (CHNS) cohort study in 2006–2011. The risk of new-onset overweight with different amounts of PWI per day was analyzed in this 5-year cohort. A multiple logistic regression model was used to assess the association of PWI and the risk of new-onset overweight and adjust for potential confounders. Moreover, dose-response models were developed to estimate the linear relationship.

**Results:**

During 5 years of follow-up, 1,018 incident cases were identified. Our analysis indicated an inverse association of more than 4 cups of PWI per day and the risk of new-onset overweight among normal weight individuals. Compared with participants who drank 2 to 3 cups PWI, the adjusted odds ratios (OR) of overweight were 0.741 (95% confidence interval [CI], 0.599–0.916) in participants who drank 4 to 5 cups PWI, and 0.547 (95% CI, 0.435–0.687) in participants who drank more than 6 cups PWI. The dose-response analysis showed that every cup of PWI was associated with a 6.5% and 8.4% decrease in the risk of new-onset overweight among men and women, respectively. The interactions of PWI and covariates on the risk of overweight were not found.

**Conclusion:**

Drinking more than 4 cups (≈1 liter) per day of plain water is associated with decrease in the risk of new-onset overweight among normal-weight individuals.

## INTRODUCTION

The rising prevalence of overweight and obesity has developed into an epidemic public problem around the world.^1^ Similar results obtained in China showed that age-adjusted prevalence of overweight (body mass index [BMI] ≥24.0 kg/m^2^) among Chinese adults has increased from 20.5% to 42.3% over a 20-year period (1991–2011).^2^ It is important to prevent overweight patients from developing obesity in future years. Hence, it is highly critical to reduce the risk of overweight among the normal-weight population to decrease the number of new-onset overweight patients.

Although many studies have estimated the relationship of beverages and weight gain risk, much less is known about plain water.^3^^,^^4^ Drinking more water has been proven as a method to reduce the risk of weight gain in randomized controlled trials (RCTs).^5^^,^^6^ As reviewed by Daniels, the findings from clinical trials, along with the intervention studies, suggested that water was potentially associated with reducing daily food and calories beverage intake, and consequently in obesity prevention. Additionally, increased water intakes could effectively decrease the risk of obesity and related diseases.^5^ Epidemiologic evidence also suggests that increasing water intake could decrease the risk of weight gain. Results from three separate large prospective cohorts of American nurses and physicians suggested that the substitution of water for sugar-sweetened beverages or fruit juices was associated with lower the risk of weight gain in each 4 years.^7^ A similar study, which was conducted among university students, showed that increasing water intake in place of sugar beverages or beers was related to a lower incidence of obesity and overweight.^8^

Nevertheless, few studies provided the population-based evidence regarding the association of PWI and the overweight risk. Additionally, most studies focused on the effect of PWI replacing caloric beverages to reduce the risk of weight gain, which ignored the independent effect of PWI on the weight gain. The aim of our study was to investigate the independent effect of PWI on the risk of new-onset overweight over time using national data (2006–2011 China Health and Nutrition Survey [CHNS]) among Chinese adults.

## MATERIALS AND METHODS

### Study design

The CHNS was conducted collaboratively by the National Institute for Nutrition and Food Safety, Chinese Center for Disease Control and Prevention, and University of North Carolina at Chapel Hill. The CHNS was initialed in 1989 and continued in 1991, 1993, 1997, 2000, 2004, 2006, 2009, and 2011 in the nine provinces (Liaoning, Jiangsu, Shandong, Henan, Hubei, Guangxi, Guizhou, and Heilongjiang) until 2011, which covered low-, middle- and high-income areas around China, accounting for approximately 56% of the Chinese population. A multistage random cluster method was used for the survey sampling design in each of the surveyed provinces. Each wave survey selected two cities (usually a provincial capital large city and a small city) and four counties based on income (one high-income, one low-income, and two middle-income counties) in each province. In each city, four communities (two urban and two suburban) were randomly chosen. In each county, four communities (one from the capital city and three rural villages) were randomly selected. Twenty households within each community were selected randomly for survey in the CHNS. The study population included in the CHNS was limited to free-living members. The detail information of the CHNS has been published elsewhere.^9^^,^^10^ The ethics review committees of the Chinese Center for Disease Control and Prevention, and University of North Carolina at Chapel Hill approved the study protocol.

### Study population

In this study, people who participated two waves survey (2006 and 2011 CHNS) were analyzed, while participants with missing data including age, BMI, and PWI (*n* = 1,484), as well as pregnant and breastfeeding participants (*n* = 52), were excluded. Further, participants were also excluded with extreme values (BMI <18.5 and ≥40.0; total energy intake ≤800 kcal/day or ≥6,000 kcal/day for man and ≤600 kcal/day or ≥4,000 kcal/day for woman; and sleep duration <6 hours/day and >12 hours/day) (*n* = 43). Considering possible confounding by disease- and metabolism-relative weight changes, we also excluded participants who had diabetes (*n* = 101), cancer (*n* = 11), weight change over ±5 kg (*n* = 11), and waist circumference change over ±10 cm (*n* = 10) at baseline in the cohort study.

### Assessment of plain water and other beverages

A frequency questionnaire and the China food composition tables (FCTs) were used to collect daily water and food intake. Data about PWI and beverage intake was collected from 2006 and 2004, respectively, using self-report in the CHNS. The question, “How many cups (1 cup ≈ 240 mL) did you drink per day?” was used for the consumption of plain or bottled water, tea, and coffee. Six similar questions were used for investigating the consumption of alcoholic beverages (grape wine, liquor, and beer) and sugar beverage (Chinese brand soft drinks [CBSDs], non-Chinese brand soft drinks [non-CBSDs], and sugared fruit drinks) intake. The questionnaires are publicly available at http://www.cpc.unc.edu/projects/china/data/questionnaires.

### Assessment of other covariates

For the 5-year cohort study, overweight-related and sociodemographic factors were also assessed: physical activities, smoking status, cigarettes consumption, sedentary behaviors, sleep duration, highest education level, household income, total energy intake, beverage intake, and dietary habits (rice, wheat, fruits, vegetables, red meats and poultry). Smoking status was categorized as past, current, and never smoked. Urbanization was categorized as urban and rural. Highest education level was categorized as low-level (primary school and lower middle school degree), middle-level (upper–middle school degree and technical/vocational degree), and high-level (university or college degree and master’s degree or higher). The metabolic equivalent index (MET) codes and the detailed questions of the physical activity survey have been published elsewhere.^11^^–^^13^ Sedentary behaviors were defined as reading, drawing, watching TV, surfing the internet, participating in chat rooms, playing computer games, and watching movies online using average time per day (hours/day). Household income was summarized by each level of income listed in the questionnaire.

Energy intake was calculated from 3-day dietary-recall Chinese FCTs. The 3-day (2 weekdays and 1 weekend day) dietary-recall FCTs were used for collecting daily food intake with a food-weighted method in each wave of the CHNS. The FCTs were used in 2006, 2009, and 2011 in the same way. The 24-h dietary recalls were administered by trained interviewers using standard forms in every household interview, and the daily energy intake was calculated by aggregating the energy contained in each food consumed daily based on FCTs. The measurement of total energy intake was validated using the doubly-labeled water method with all assays undertaken in the Human Nutrition Research Center of Tufts University. The correlation coefficient between the two methods was 0.56 for men and 0.60 for women.^14^

### Assessment of outcome

The weight and height of participants were measured based on the World Health Organization standard. Normal weight was defined as BMI <24.0 kg/m^2^, and overweight was defined as BMI ≥24.0 kg/m^2^, according to the criteria of the Working Group on Obesity in China (WGOC).^15^ The participants who were normal weight were followed up from 2006 and the participants who were overweight were identified in 2011.

### Statistical analysis

All continuous variables were presented as mean (fifth percentile, ninety-fifth percentile) at baseline to obtain more information about various changes in differences between individuals.^7^ Mann-Whitney test was used for continuous variables analysis. All analysis was performed using Stata, version 12.0 (Stata Corporation, College Station, TX, USA). A two-tailed *P*-value <0.05 was considered statistically significant.

Multiple logistic regression models were used to assess the risk of new-onset overweight compared to PWI (0–1 cup/day, 2–3 cups/day, 4–5 cups/day, and ≥6 cups/day). The odds ratios (ORs) and 95% confidence intervals (CIs) were used as a measure of the association between PWI and overweight risk. Considering various consumption of PWI in different regions and household, 95% CI was calculated using robust clustered standard error to reduce the effect of intra-cluster correlation.^16^^,^^17^ Only baseline PWI was used to estimate the risk of overweight, and the between-persons differences of PWI were non-significant during 2006 and 2011 CHNS (*P* > 0.05). To estimate the effect of lifestyle and diet habits, model 2 adjusted for the age and baseline BMI, urbanization, and education level as well as changes in cigarettes consumption, smoke status, sedentary time, sleep duration, and household income. Model 3 and model 4 further adjusted Chinese dietary habits and beverages to assess the effect on the risk of weight gain, respectively. Finally, model 5 adjusted the total energy intake and physical activity. Since tea is popular in China, especially among older adults,^18^ tea was counted in PWI to estimate the risk of overweight in different age groups using the same regression model.

Dose-response models were used to assess the linear relationship between PWI and new-onset overweight risk by the generalized least squares method.^19^^,^^20^ The log-linear dose-response model and the random-effect non-linear dose-response model was developed to examine the effect of PWI on decreased risk of overweight. This method of analysis has been widely applied in the dose-response analysis.^21^

Subgroup and sensitivity analyses were conducted stratified by potential effect modifiers, including tea consumption, cigarette consumption, alcohol intake, sugar beverage intake, urbanization, physical activity, total energy intake, household income, and sedentary time. The risk of overweight was estimated in high dose group and low dose group, which was divided by the median consumption. The interaction was assessed by including each term and its cross-product terms together in multiple adjusted models. For example, the term of sleep duration, PWI, and sleep duration × PWI were included together in the model for the interaction analysis. To test the robustness of our results, we excluded participants with boundary BMI values (23.0 ≤ BMI ≤ 23.9 kg/m^2^) (*n* = 89), who had greater chance of becoming overweight.

## RESULTS

### Baseline characteristics of participants

**Figure 1.**
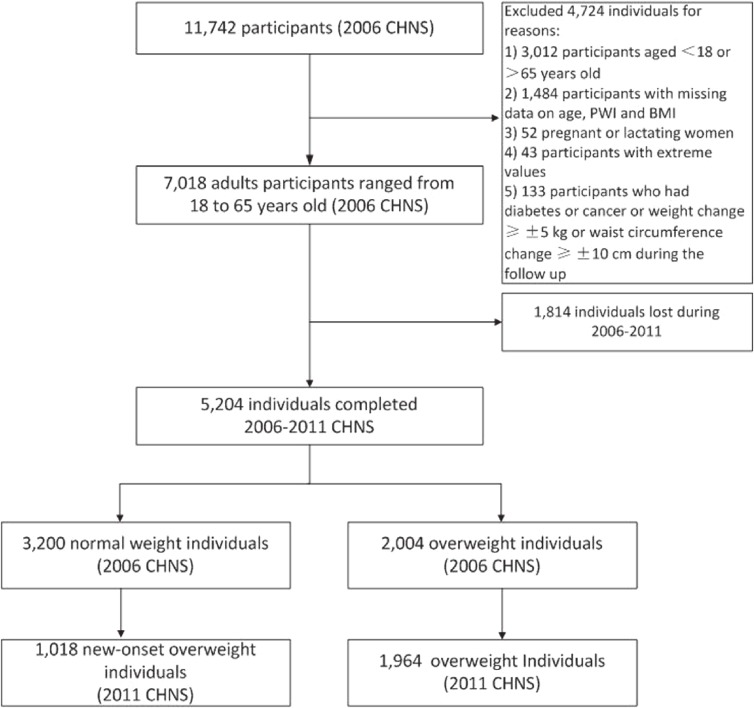
Flow chart illustrating the sample selection for the present study.

A total of 7,018 participants, 3,353 men and 3,665 women were enrolled in 2006 CHNS, ranging from 18 to 65 years old. There were 5,204 participants (about 74%) who completed 2006–2011 CHNS. The flow chart of enrolled participants in our study is shown in Figure 1. 2006 CHNS demographics and beverage behaviors stratified by gender with mean (fifth percentile, ninety-fifth percentile) are presented in the Table 1. The mean of PWI was 582 mL/day among men and 574 mL/day among women at baseline. Plain water and tea intake accounted for most part of daily fluid intake. Additionally, the distribution of other beverages intake among groups stratified by water intake is displayed in eTable 1, which reveals that beverage intake except for tea was relatively evenly distributed in different PWI groups. Furthermore, when the 51- to 65-year-old participants were excluded, the tea intake was also evenly distributed in the plain water intake groups (eTable 2).

**Table 1.** Baseline and change of characteristics among 3,200 normal weight participants in the 5-year cohort: CHNS 2006–2011^a^

Variables		Men (*n* = 1,498)		Women (*n* = 1,702)	
		**Baseline**	**Change within 5 years**	**Baseline**	**Change within 5 years**
**Age, years**		45.8 (23.9, 63.0)	—	45.0 (27.0, 62.0)	—
**BMI, kg/m^2^**		21.3 (18.1, 23.7)	0.15 (−3.5, 4.6)	20.9 (17.9, 23.1)	0.07 (−1.1, 1.6)
**Physical activity, MET-hours per week**		284 (23, 358)	−85 (−99, 217)^b^	271 (10, 335)	−70.2 (−101, 205)^b^
**Energy intake**		2,010 (1,449, 5,031)	54 (−235, 1,115)^b^	2,008 (1,856, 4,889)	64 (−201, 1,203)^b^
**Household income, thousand Yuan/year**		9 (1.0, 40.1)	9.7 (0.4, 10.3)^b^	9 (1.5, 45.2)	9.9 (1.2, 10.4)^b^
**Sleep duration, hours/d**		8 (6, 10)	0 (−3, 5)	8 (5, 10)	0 (−3, 5)
**Sedentary behaviour, hours/week**		33 (0, 80)	0.7 (−5.0, 4.0)	34 (0, 80)	1.8 (−7.4, 5.6)
**Cigarettes consumption, packs/d**		5 (0, 20)	0.3 (−10, 12)	0 (0, 9)	0 (−3, 5)
**Urbanization**					
	Urban	515	—	606	—
	Rural	983		1,096	
**Education level**					
	Low	1,031	—	1,172	—
	Middle	294	—	334	—
	High	173	—	196	—
**Smoke status**					
	Past	415	—	50	—
	Current	328	—	46	—
	Never	755	—	1,606	—
**Beverages intake, mL per day**					
**Plain water**		582 (0, 1,440)	170 (−240, 480)	574 (0, 1,440)	161 (−240, 480)
**Tea**		364 (0, 1,250)	76 (−480, 480)	175 (0, 1,200)	43 (−480, 480)
**Coffee**		6 (0, 24)	17.4 (0, 24)^b^	5 (0, 24)	10.2 (0, 24)^b^
**CBSDs**		6 (0, 14)	9.6 (−15.4, 27.8)^b^	6 (0, 15)	10.4 (−14.1, 28.0)^b^
**Non-CBSDs**		8 (0, 14)	4.8 (0, 27.9)	11 (0, 14)	6.6 (0, 28.4)
**Sugared fruit drinks**		10 (0, 71)	10.0 (−14.3, 28.6)^b^	11 (0, 64)	14.8 (−13.2, 30.1)^b^
**Group wine**		4 (0, 4)	2.4 (0, 4.6)^b^	1 (0, 2)	0.4 (0, 1.5)^b^
**Liquor**		48 (0, 72)	−2.6 (−28.5, 23.0)	1 (0, 60)	0 (−5.3, 5.0)
**Beer**		97 (0, 215)	−0.3 (−25.0, 25.0)	10 (0, 220)	−9.8 (−25.0, 25.0)^b^
**Dietary intake, g per day**					
**Rice**		318 (0, 600)	−35.1 (−70.1, 11.3)	306 (0, 600)	−34.6 (−69.0, 12.1)
**Wheat**		186 (0, 466)	−34.4 (−68.6, 9.3)^b^	170 (0, 510)	−33.9 (−59.4, 8.9)^b^
**Fruits**		45 (0, 308)	29.9 (−11.7, 42.3)^b^	47 (0, 350)	31.1 (−11.5, 41.6)^b^
**Vegetables**		300 (76.9, 650)	24.6 (−9.8, 33.2)	306 (80, 667)	25.2 (−9.9, 35.8)
**Red meats**		61 (0, 200)	9.8 (−25.6, 36.6)^b^	62 (0, 200)	9.4 (−23.4, 35.2)^b^
**Poultry**		9 (0, 100)	8 (−23.2, 37.8)^b^	9 (0, 100)	8 (−22.2, 33.7)^b^

BMI, body mass index; MET, metabolic equivalence task; CBSDs, Chinese brand soft drinks; CHNS, China Health and Nutrition Survey; non-CBSDs, non-Chinese brand soft drinks.

^a^Data were presented as mean (fifth percentile, ninety-fifth percentile).

^b^*P*-value <0.05.

#### Risk of plain water intake and overweight: CHNS 2006–2011

There were 1,018 new-onset incident cases (about 31.8%) were identified in the normal-weight group at baseline and 1,964 participants (about 98%) were still overweight during the follow up. To be closer to the real world condition, we identified the daily 2- to 3-cup PWI group as reference which covered the most participants. Compared to the daily 2- to 3-cup PWI group, the risk of new-onset overweight decreased 25.9% and 45.3% in the 4- to 5-cup group and in ≥6-cup group, respectively, after adjusting all overweight-related confounders. Furthermore, the results showed that ≥6 cups daily of PWI was associated with an even more reduced risk of overweight in women (OR 0.509; 95% CI, 0.379–0.683) than in men (OR 0.557; 95% CI, 0.422–0.735) (Table 2). Despite the different extents of risk decrease in different gender groups, the interaction was not statistically significant (*P*_interaction_ > 0.05).

**Table 2.** Odds ratio (OR) and 95% confidence intervals (CIs) for incidence of new-onset overweight associated with consumption of plain water intake per day in the 5-year period cohort: CHNS 2006–2011

Variables		Cups/day	*N* (%)	Cases^a^	OR (95% CI)				

					Model 1^b^	Model 2^c^	Model 3^d^	Model 4^e^	Model 5^f^
**Total**			3,200 (100)	1,018					
		0–1	684 (21.4)	221	0.874 (0.809, 0.944)	0.887 (0.719, 1.094)	0.902 (0.730, 1.114)	0.883 (0.721, 1.081)	0.883 (0.721, 1.081)
		2–3	1,257 (39.3)	444	Reference	Reference	Reference	Reference	Reference
		4–5	913 (28.5)	270	0.769 (0.633, 0.933)	0.744 (0.617, 0.896)	0.743 (0.616, 0.896)	0.750 (0.622, 0.904)	0.741 (0.599, 0.916)
		≥6	346 (10.8)	83	0.578 (0.453, 0.736)	0.534 (0.405, 0.705)	0.530 (0.401, 0.701)	0.540 (0.409, 0.713)	0.547 (0.435, 0.687)
	*P*-value				<0.0001	<0.0001	<0.0001	<0.0001	<0.0001
**Gender**									
	**Men**		1,498 (100)	466					
		0–1	338 (22.6)	101	0.768 (0.684, 0.861)	0.791 (0.578, 1.083)	0.818 (0.596, 1.121)	0.777 (0.575, 1.051)	0.777 (0.575, 1.051)
		2–3	549 (36.6)	196	Reference	Reference	Reference	Reference	Reference
		4–5	439 (29.3)	124	0.709 (0.523, 0.954)	0.654 (0.494, 0.866)	0.655 (0.494, 0.869)	0.676 (0.511, 0.894)	0.654 (0.485, 0.883)
		≥6	172 (11.5)	45	0.638 (0.473, 0.862)	0.532 (0.357, 0.792)	0.537 (0.360, 0.802)	0.560 (0.377, 0.833)	0.557 (0.422, 0.735)
	*P*-value				0.011	0.003	0.003	0.007	0.007
	**Women**		1,702 (100)	552					
		0–1	346 (20.3)	120	0.985 (0.782, 1.240)	0.972 (0.727, 1.299)	0.999 (0.745, 1.340)	1.013 (0.766, 1.339)	0.977 (0.782, 1.221)
		2–3	708 (41.6)	248	Reference	Reference	Reference	Reference	Reference
		4–5	474 (27.8)	146	0.826 (0.727, 0.938)	0.816 (0.632, 1.052)	0.813 (0.628, 1.051)	0.820 (0.634, 1.059)	0.822 (0.716, 0.943)
		≥6	174 (10.2)	38	0.518 (0.383, 0.701)	0.512 (0.344, 0.763)	0.506 (0.339, 0.756)	0.515 (0.345, 0.768)	0.509 (0.379, 0.683)
	*P*-value				0.008	0.007	0.005	0.006	0.006

BMI, body mass index; CHNS, China Health and Nutrition Survey; *N*, number of participants.

^a^Number of participants who developed overweight within the five-year follow-up.

^b^Model 1: Unadjusted OR.

^c^Model 2: Adjusted for the age and baseline BMI, urbanization, education level, smoke status as well as changes in cigarettes consumption, sedentary time, sleep duration, and household income.

^d^Model 3: Adjusted for all variables in Model 2 as well as changes in physical activity, rice, wheat, fruits, vegetables, all red meats and its products, and poultry.

^e^Model 4: Adjusted for all variables in Model 2 as well as changes in physical activity, intake of tea, coffee, CBSDs, non-CBSDs, sugar juice, grape wine, liquor and beer.

^f^Model 5: Adjusted for all variables in Model 2 as well as changes in physical activity and total energy intake.

**Figure 2.**
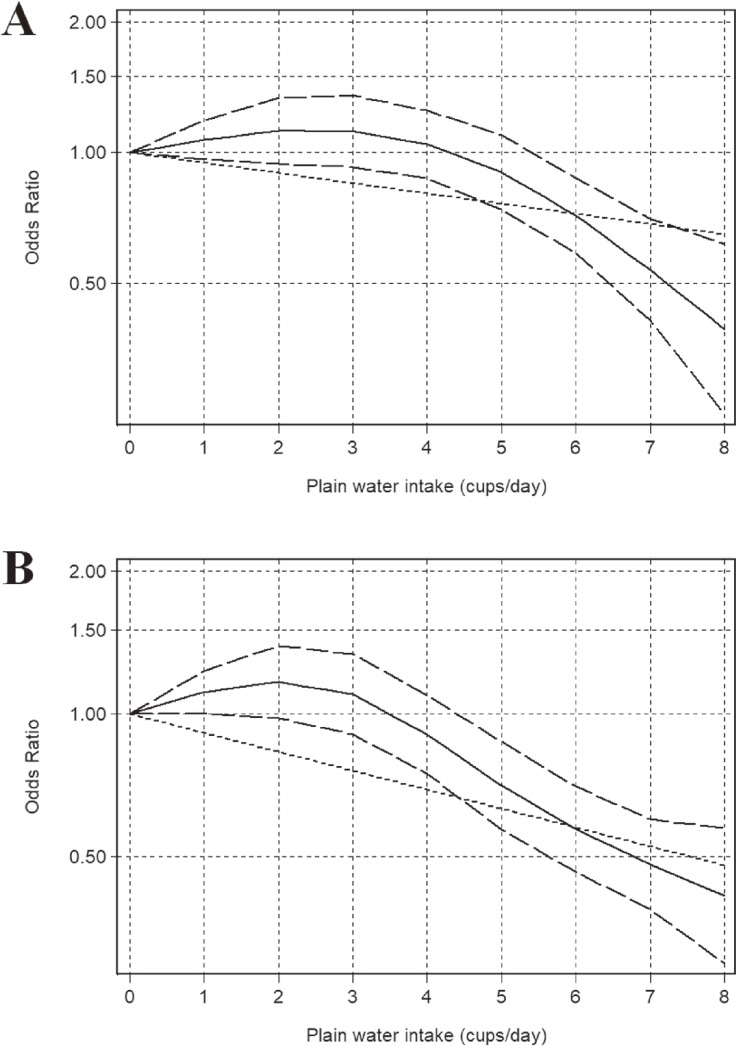
Dose-response relationship of plain water intake and the risk of overweight among men (A) and women (B). Log-linear dose-response model was present by odds (OR: - - - - -). Odds ratios (OR: ——) and corresponding 95% CI (— —) were summarized for the non-linear dose-response model relationship between plain water intake and risk of overweight.

There was a dose-response relationship between PWI increase and the risk of new-onset overweight among normal weight individuals (Figure 2). According to the dose-response models, the risk of new-overweight decreased 6.5% among men (A) and 8.4% among women (B) with each cup increment per day under the linear model. Moreover, in the non-linear dose-response relationship, there was a significant trend towards a reduction in new-onset overweight risk when the PWI was ≥6 cups per day (*P*_non-linearity_ < 0.001).

**Figure 3.**
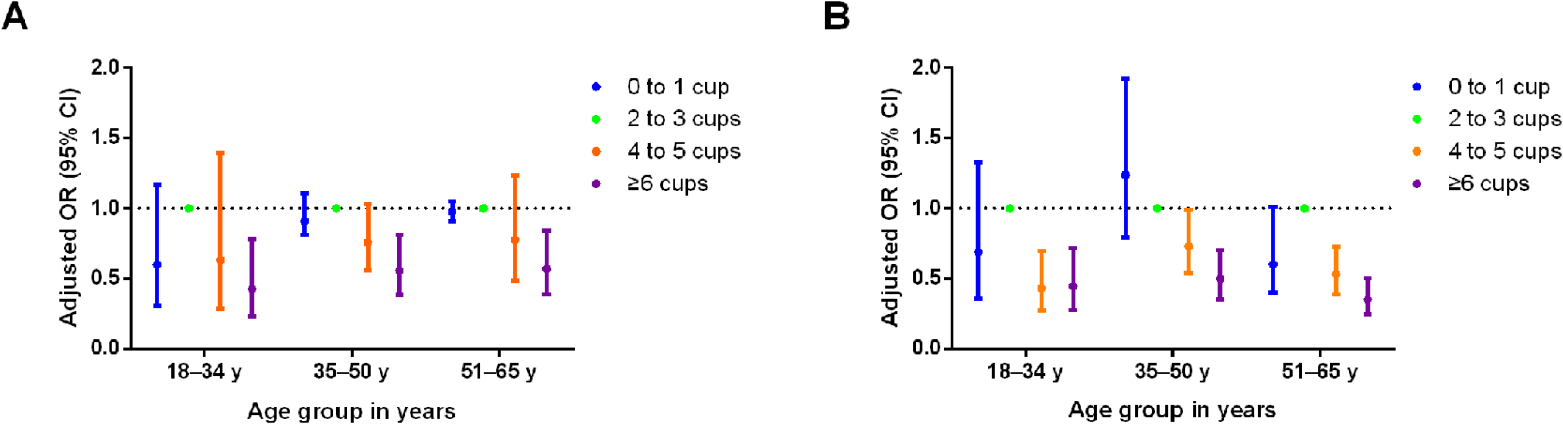
The odds ratios and corresponding 95% confidence interval for new-onset overweight risk for consumption of PWI (A) and PWTI (B).

Again, the association of PWI and plain water and tea intake (PWTI) and new-onset overweight risk is shown in Figure 3A and Figure 3B, respectively. The risk of overweight was not obviously different among various age groups from among total participants. Although the risk of overweight with more than 4 cups per day PWTI was slightly lower than plain water only, the interaction of tea and PWI was not observed in the interaction analysis.

### Subgroup and sensitivity analysis

In the subgroup analysis, lower consumption of tea, cigarettes, and alcohol was associated with greater reduction of risk of overweight compared to the higher consumption group. Additionally, the association was more pronounced among participants with higher sugared-beverage intake, physical activity, total energy intake, household income, sedentary time, and urban living. However, there were no significant interaction between these covariates and PWI. The OR and 95% CI showed no material difference after excluding the subjects with boundary overweight BMI in the sensitivity analysis (Table 3).

**Table 3.** Subgroup and sensitivity analysis of OR (95% CI) for incidence of overweight associated with the daily plain water intake^a^

Variables	Daily plain water intake				*P*_trend_^b^	*P*_interaction_^c^

	0–1 cup	2–3 cups	4–5 cups	≥6 cups		
**Overall**	0.883 (0.721, 1.081)	Reference	0.741 (0.599, 0.916)	0.547 (0.435, 0.687)	<0.001	
**Tea consumption**						
Less than 4 cups	0.929 (0.745, 1.160)	Reference	0.724 (0.595, 0.880)	0.526 (0.393, 0.705)	<0.001	
More than 4 cups	0.887 (0.523, 1.502)	Reference	1.038 (0.560, 1.923)	0.823 (0.344, 1.971)	0.929	0.484
**Cigarettes consumption**						
Low	0.909 (0.672, 1.229)	Reference	0.728 (0.565, 0.938)	0.508 (0.346, 0.745)	0.002	
High	0.937 (0.710, 1.236)	Reference	0.747 (0.568, 0.985)	0.607 (0.407, 0.907)	0.036	0.724
**Alcohol intake**						
Low	0.928 (0.685, 1.256)	Reference	0.743 (0.577, 0.957)	0.515 (0.351, 0.755)	0.003	
High	0.918 (0.696, 1.210)	Reference	0.751 (0.570, 0.989)	0.603 (0.404, 0.901)	0.039	0.566
**Sugar beverages intake**						
Low	0.893 (0.667, 1.196)	Reference	0.847 (0.658, 1.091)	0.579 (0.387, 0.865)	0.058	
High	0.923 (0.694, 1.227)	Reference	0.636 (0.482, 0.838)	0.519 (0.354, 0.760)	<0.001	0.241
**Urbanization**						
Urban	0.957 (0.564, 1.625)	Reference	0.598 (0.375, 0.952)	0.470 (0.238, 0.928)	0.048	
Rural	0.964 (0.735, 1.263)	Reference	0.816 (0.643, 1.035)	0.479 (0.329, 0.698)	0.001	0.884
**Physical activity**						
Low	0.859 (0.636, 1.160)	Reference	0.750 (0.575, 0.980)	0.511 (0.343, 0.763)	0.006	
High	0.991 (0.726, 1.353)	Reference	0.723 (0.551, 0.949)	0.499 (0.331, 0.753)	0.002	0.691
**Total energy intake**						
Low	0.898 (0.672, 1.201)	Reference	0.821 (0.633, 1.065)	0.631 (0.428, 0.932)	0.102	
High	0.924 (0.697, 1.226)	Reference	0.718 (0.551, 0.937)	0.501 (0.338, 0.742)	0.002	0.523
**Household income**						
Low	0.987 (0.729, 1.337)	Reference	0.735 (0.556, 0.973)	0.559 (0.370, 0.843)	0.013	
High	0.851 (0.626, 1.157)	Reference	0.705 (0.543, 0.915)	0.449 (0.302, 0.667)	<0.001	0.528
**Sedentary time**						
Low	0.967 (0.706, 1.325)	Reference	0.653 (0.498, 0.856)	0.640 (0.431, 0.949)	0.006	
High	0.903 (0.671, 1.215)	Reference	0.799 (0.611, 1.044)	0.402 (0.266, 0.607)	<0.001	0.325
**Excluded participants with boundary BMI value (BMI: 23.0–23.9)**	0.948 (0.771, 1.166)	Reference	0.776 (0.630, 0.932)	0.582 (0.434, 0.782)	0.001	

^a^The model adjusted the age and baseline BMI, urbanization, education level, smoke status as well as changes in cigarettes consumption, sedentary time, sleep duration, household income and total energy intake.

^b^*P*_trend_, *P*-value for trend.

^c^*P*_interaction_, *P*-value for interaction.

## DISCUSSION

To our knowledge, there have been few studies reporting the independent effect of PWI on weight gain and the incidence of overweight among large free-living population. During 5-year follow up, we found that more than 4 cups per day PWI was significantly and inversely associated with the incidence of overweight among Chinese adults. Similar results were observed among men and women, as well as among all age groups. An inverse dose-response relationship between PWI and the risk of overweight showed that every cup of PWI was associated with a 6.5% and 8.4% decrease in the risk of new-onset overweight among men and women, respectively. The association of PWI and overweight was independent of socioeconomic and lifestyle factors, such as age, gender, physical activities, smoking status, cigarettes consumption, sedentary behaviors, sleep duration, highest education level, household income, and total energy intake.

Our findings with regard to PWI and weight gain are consistent to several RCT studies. A famous A to Z RCT study showed that individuals who drank >1 liter water per day had greater losses in weight (2.3 kg) and waist circumference (2.3 cm) over 12 months, and a reduction of 0.03 kg weight, 0.03 cm waist circumference, and 0.02% body fat per day if 1% sugared calories beverages were replaced with plain water among 173 premenopausal overweight female adults, despite diet and physical activity.^22^ Interestingly, our findings showed that more than 4 cups (≈1 liter) per day PWI was associated with lowering the risk of new-onset overweight. In addition, metabolism studies also support our results, showing that increasing water intake could lower energy intake, increasing energy expenditure and fat oxidation to decrease weight gain or increase weight loss in the RCT condition.^5^ Another study showed that 500 mL (≈2 cups) plain water increased energy expenditure by 100 KJ.^23^ For epidemiology studies, Pan et al found that each 1 cup per day increment of plain water was associated with weight gain −0.13 (95% CI, −0.17 to −0.08) kg over each 4-year period after adjusting for age, baseline BMI and changes in diet behaviors and physical activity. Additionally, substitution of plain water with sugared caloric beverages was associated with 0.49 (95% CI, 0.32 to −0.65) kg weight gain during the 4-year follow-up.^7^ A similar cohort study conducted in university students showed that the consumption of plain water instead of sugared caloric beverages was associated with 20% and 15% lower risk of obesity and overweight, respectively, as well as −205 g weight gain within the 4-year follow-up after controlling physical activity and energy intake.^8^ As our study reported, the risk of overweight would decrease 25.9% and 45.3% by consuming 4 to 5 cups and ≥6 cups of plain water compared to daily intake of 2 to 3 cups, after adjusting all overweight-related confounders. The lower BMI threshold value of overweight and smaller body size may lead to more risk reduction among Chinese population compared to American and European population. However, the Chinese population may be more appropriate to study the relationship of water intake and weight change. Given studies suggesting that PWI accounted for approximately 60% of the total water intake (sum of water from all foods and all beverages) per day among the Chinese population, which was higher than the proportion among the American population of 33% in daily total water intake according to the 2005–2006 National Health and Nutrition Examination Survey (NHANES).^24^^,^^25^ Furthermore, lower-sugar caloric beverage intake habits may have less influence on PWI among the Chinese population than among American and European population.

Plain tea was popular and made up a large part of daily fluid intake in Chinese adults, especially in older individuals. Additionally, tea was recommended by the Chinese Dietary Nutrition Guidelines.^26^ Hence, tea intake was regard as PWI to re-estimate the risk of overweight in the all age groups. As reported, oolong tea^27^ and caffeinated tea^28^ increase fat oxidation, reduce body fat, and promote weight loss because caffeine may stimulate the sympathetic nervous system to increase basal energy expenditure and thermogenesis.^29^^,^^30^ Although plain tea could be recommended in the older population, our analysis did not show the risk changes after considering tea as plain water, and there was no statistical significance between high and low tea intake groups in subgroup analysis. Disproportionate distribution of tea type and various drinking habits among participants may be linked to the results. As we know, older Chinese people preferred to drink strong tea instead of plain water, but the younger Chinese people barely like to drink tea. This reason may explain why more changes of OR in the older groups after regarding tea as plain water.

In conclusion, most clinical studies focused on the intervention of weight loss, instead of the prevention of weight gain at present. Although interventions for obesity patients have been conducted for several years, the prevalence of overweight and obesity is still increasing every year around the world.^1^ Hence, policies need to lean towards reducing the risk of weight gain among the normal-weight population and preventing them from developing overweight.

A major strength of our research was the fixed cohort study design, which basically avoids the influence of the age-period-cohort effect.^31^^,^^32^ The nationwide changes over time may affect weight gain among all population subgroups and promote changes to dietary habits, life behaviors, and the obesogenic environment. Second, to our knowledge, our study has provided the first evidence of the independent effect of PWI on decreasing the overweight risk, which will attract attention of researchers to the effect of plain water on weight management. Furthermore, our study may provide the evidence in support of Chinese PWI policies. Finally, our study is the first to use a cup of plain water as the unit to estimate the risk of overweight, which was closer to the real-world situation and easier to be adopted by the public.

This study also has some potential limitations. First, the consumption of plain water was self-reported, which may introduce bias. However, the frequency questionnaires items were strictly validated in each wave survey, which had high validity and responsibility of items in the questionnaires (https://www.cpc.unc.edu/projects/china/data/questionnaires). And in the process of analysis, the intraclass correlation coefficient (ICC = 0.72, *P* < 0.01 in men; ICC = 0.75, *P* < 0.01 in women) of the plain water intake between baseline (2006 CHNS) and 5-year follow-up (2011 CHNS) was calculated. Additionally, sensitivity analyses were conducted in different populations (including participants who only drink plain water or tea, participants who only drink plain water, as well as participants excluded at baseline), and the results were similar with the original. Both of those indicated that the results were stable and credible. Second, the baseline PWI information was used to estimate the risk of overweight, which ignored the dynamic change of individual PWI habit. This minor error is unlikely to influence our results because there was small difference between personal PWI within 5 years (fifth percentile: −240, ninety-fifth percentile: 480). Third, although a series of covariates have been controlled, PWI may be influenced by other lifestyle factors that were not measured in this survey. Finally, considering the effects of basic metabolic rate or metabolic disorders on water intake, participants who had diabetes, cancer, weight change over ±5 kg, waist circumference changes over ±10 cm at baseline, pregnant and lactating woman, participants with extreme values (including BMI, total energy intake, and sleep duration) were excluded, while the basic metabolic rate (BMR) was not measured and the effect of other metabolic disorders on water intake was not considered in this study.

### Conclusions

Drinking more than 4 cups of plain water was associated to lower risk of overweight among normal weight individuals. There is an inverse dose-response relationship between PWI and the risk of overweight.

## APPENDIX A. SUPPLEMENTARY DATA

eTable 1. Description of beverage consumption per day based on PWI among 3,200 Chinese adults aged 18 to 65 years old

eTable 2. Description of beverage consumption per day based on PWI among 1,982 Chinese adults aged 18 to 50 years old
