# Supplementary material for: Plain Water Intake and Association With the Risk of Overweight in the Chinese Adult Population: China Health and Nutrition Survey 2006–2011
Source: J Epidemiol. 2020 Mar 5;30(3):128–35. doi: 10.2188/jea.JE20180223 (PMC7025921; doi:10.2188/jea.JE20180223)
Supplement: Supplementary file 1 [file je-30-128-s001.pdf]

eTable 1. Description of beverage consumption per day based on PWI among 3,200 Chinese adults aged 18 to 65 years old

Variables	Plain water intake			
	**0–1 cup**	**2–3 cups**	**4–5 cups**	**≥6 cups**
		Men		
**Coffee**	8 (1.4)	5 (1.4)	5 (1.4)	7 (1.9)
***Tea**	596 (18.9)	450 (10.6)	255 (10.7)	279 (20.6)
**CBSDs**	5 (1.3)	8 (1.2)	8 (1.9)	9 (2.0)
**Non-CBSDs**	7 (1.5)	8 (1.3)	7 (1.8)	8 (1.8)
**Sugared**	12 (0.9)	12 (1.7)	12 (1.8)	9 (2.2)
**Grape wine**	5 (1.2)	5 (0.6)	4 (0.8)	5 (1.2)
**Liquor**	49 (1.6)	48 (1.3)	45 (1.5)	48 (2.4)
**Beer**	86 (1.1)	98 (3.6)	87 (4.3)	98 (8.3)
		Women		
**Coffee**	5 (1.7)	4 (1.3)	4 (2.1)	5 (3.5)
***Tea**	427 (22.2)	348 (6.3)	143 (7.9)	161 (14.5)
**CBSDs**	6 (1.6)	6 (1.6)	6 (2.4)	5 (2.9)
**Non-CBSDs**	13 (1.5)	10 (1.5)	13 (1.8)	11 (1.8)
**Sugared**	11 (1.4)	9 (1.6)	10 (1.8)	15 (2.4)
**Grape wine**	1 (0.3)	1 (0.1)	1 (0.2)	1 (0.2)
**Liquor**	1 (0.2)	1 (0.2)	1 (0.2)	1 (0.2)
**Beer**	8 (1.6)	6 (0.7)	7 (1.3)	12 (2.8)

CBSDs, Chinese brand soft drinks; non-CBSDs, non-Chinese brand soft drinks; PWI, plain water intake.

Data was presented as mean (standard error). * p-value<0.05.

eTable 2. Description of beverage consumption per day based on PWI among 1,982 Chinese adults aged 18 to 50 years old

Variables	Plain water intake			
	**0–1 cup**	**2–3 cups**	**4–5 cups**	**≥6 cups**
		Men		
**Coffee**	7 (2.1)	5 (1.8)	6 (1.9)	7 (2.1)
**Tea**	284 (19.3)	261 (12.4)	242 (13.8)	277 (22.3)
**CBSDs**	5 (1.8)	7 (1.7)	8 (2.3)	9 (2.5)
**Non-CBSDs**	7 (1.9)	7 (1.5)	7 (1.7)	8 (2.1)
**Sugared**	13 (1.4)	12 (2.0)	11 (2.2)	9 (2.8)
**Grape wine**	5 (1.8)	4 (1.4)	4 (1.6)	4 (1.9)
**Liquor**	50 (2.8)	49 (2.1)	43 (1.9)	48 (2.7)
**Beer**	88 (1.5)	88 (3.5)	97 (5.1)	96 (10.1)
		Women		
**Coffee**	5 (2.1)	4 (1.7)	4 (2.3)	4 (4.0)
**Tea**	182 (23.5)	155 (10.5)	161 (12.7)	175 (18.6)
**CBSDs**	6 (2.2)	6 (2.4)	6 (2.7)	5 (3.3)
**Non-CBSDs**	12 (1.8)	13 (1.8)	12 (1.8)	11 (2.4)
**Sugared**	11 (2.0)	10 (1.9)	10 (2.2)	12 (2.9)
**Grape wine**	1 (0.6)	1 (0.2)	1 (0.3)	1 (0.2)
**Liquor**	1 (0.2)	1 (0.2)	1 (0.2)	1 (0.2)
**Beer**	8 (1.6)	6 (0.7)	7 (1.3)	12 (2.8)

CBSDs, Chinese brand soft drinks; non-CBSDs, non-Chinese brand soft drinks; PWI, plain water intake.

Data was presented as mean (standard error). * p-value<0.05.
